# Entrepreneurial Team Knowledge Diversity and Creativity: A Multilevel Analysis of Knowledge Sharing, Individual Creativity, and Team Creativity

**DOI:** 10.3389/fpsyg.2021.717756

**Published:** 2021-09-01

**Authors:** Fei Hou, Yu Su, Mingde Qi, Lihua Wang, Qian Wang

**Affiliations:** ^1^Institute of Advanced Studies in Humanities and Social Sciences, Beijing Normal University, Zhuhai, China; ^2^Management School, Guangdong University of Technology, Guangzhou, China

**Keywords:** entrepreneurial team knowledge diversity, team creativity, knowledge sharing, sequential mediation, multilevel analysis

## Abstract

Although the academic community has consistent with the key role of entrepreneurial team knowledge diversity (ETKD), which serves as a critical catalyst of creativity in organizations, the extant research on the link between knowledge diversity and creativity is mainly concerned with individual creativity in single-level analyses. With emerging entrepreneurial ventures increasingly relying on innovation enhancement in the form of teams, there is research motivation to explore how team-level creativity develops. In this sense, this study attempts to investigate the underlying mechanism through which ETKD is associated with team-level creativity. Through a multilevel mediation model, this study proposes that ETKD can facilitate team creativity (TC) sequentially transmitted through individual-level team members' knowledge sharing (KS) and creativity. Based on a survey of 252 team members from 42 entrepreneurial teams in China, multilevel structural equation modeling (MSEM) is applied to test the top–down relationship between ETKD and KS, as well as the bottom-up link between individual creativity and TC. The findings show that our hypotheses are supported. Our findings provide some of the first empirical evidence to examine how knowledge-based diversity of entrepreneurial teams facilitates TC potential by multilevel approach. Theoretical contributions and practical implications are also offered.

## Introduction

In recent years, an increasing number of entrepreneurial firms have typically been operated not solely by entrepreneurs but rather by entrepreneurial teams. Microsoft, Google, and Apple as US cases and Alibaba and Huawei as China cases all provide anecdotal evidence to support the growing body of empirical findings. Increasing evidence shows that it is a widespread phenomenon for the foundation and operation of new ventures to be headed by entrepreneurial teams (e.g., Feeser and Willard, 1990), particularly in high-tech industries (Francis and Sandberg, 2000). However, entrepreneurial teams have been widely neglected as a stream of research by prior studies.

Although previous studies offer a variety of plausible arguments for the potential benefits of entrepreneurial teams, whether and how entrepreneurial firms' performance would be impacted are not clarified. On the one hand, many studies show that startups in the form of entrepreneurial teams are on average more successful than those in the form of solo entrepreneurs (e.g., Roberts, 1991). In this respect, entrepreneurial teams can facilitate the development of new technologies and turn ideas into commercial systems and programs through team members' interaction and cooperation (Bygrave and Hofer, 1992). On the other hand, other studies fail to provide evidence for the team–success relationship (Doutriaux, 1992; Der Foo et al., 2005). Thus, these mixed results indicate that entrepreneurial firms in the form of team foundations might be one of the key conditions of venture success. The results further suggests that the interaction among team members could facilitate the performance of new entrepreneurial firms in the process of new product or service development.

In this regard, much research effort has focused on the relationship between diversity and team creativity (TC), with the increasing popularity of working teams and the growing importance of organizational creativity (van Knippenberg and Hoever, 2017). However, prior studies remain controversial regarding the implications of team diversity, and the cliché of a double-edged sword prevails in terms of membership diversity (Bell et al., 2011; Pieterse et al., 2013). In light of the information processing view or value-in-diversity perspective, team performance could be enhanced by membership diversity via offering human capital with diverse resources, which could lead to challenging task success (Hülsheger et al., 2009). In particular, the knowledge-based diversity of teams can facilitate organizational outcomes, including TC, by integrating a pool of resources (Williams and O'Reilly, 1998). In contrast, as advanced via social categorization theory, member diversity could arouse biased perceptions and discriminations, called social categorization processes, which might counteract the diversity benefits. In this vein, team members with various social backgrounds block intrateam communication and joint efforts toward collective performance (van der Vegt, 2002; Cunningham and Sagas, 2004; Harrison and Klein, 2007). Given that the current literature has mixed ends about this relationship (Shin and Zhou, 2007; Hülsheger et al., 2009; van Knippenberg and Hoever, 2017; Park et al., 2018), these deficiencies necessitate a call for more research on the diversity–TC link (Wang et al., 2016).

Owing to the inequal effects of all types of team diversity (e.g., Milliken and Martins, 1996), examining the specific type of diversity that is most associated with outcomes of interest has been advocated (Shin and Zhou, 2007). Given that knowledge-based diversity is considered the most salient and important for generating new ideas (Nijstad and Paulus, 2003), the current study answers the call by focusing on knowledge-based diversity and exploring how a team's knowledge heterogeneity is associated with a team's creativity. Furthermore, most prior studies on knowledge-based diversity have been conducted almost exclusively within top management teams (TMTs) in mature firms (Milliken and Martins, 1996) or R&D teams (Nijstad and Paulus, 2003; Shin and Zhou, 2007) instead of other forms of teams. In contrast, a scarce body of research in the field of knowledge-based diversity pays attention to entrepreneurial teams. Following Wiersema and Bird (1993) suggestion, considering that how team members are sensitive and respond to their diversity may be contingent on the context, one should be cautionary in applying the results from one kind of team to another kind of team. Therefore, this study focuses on entrepreneurial teams and explores how knowledge-based diversity contributes to a team's subsequent creativity potential.

To explore the link between the demographic heterogeneity of teams and the related organizational outcomes, the exclusive focus on the direct effects of team diversity has been insufficient in interpreting the underlying mechanism due to omitting the intervening variables (e.g., team decision-making, team conflict) and related inferences (Henneke and Lüthje, 2007). Knowledge sharing (KS) among team members has been identified as a critical intervening process to further explore the relationship between diversity and creativity based on the fact that knowledge-sharing and knowledge-exchange are core catalysts of TC potential (Taylor and Greve, 2006; Zhou et al., 2009; Sung and Choi, 2019). However, the diverse literature has omitted knowledge-related processes because prior prevailing research attention has mostly paid to interpersonal challenges (e.g., stereotyping, dissent, and conflict; van der Vegt, 2002; Cunningham and Sagas, 2004), which have been considered of limited value-adding importance when examining positive team outcomes, especially creativity (van Knippenberg et al., 2004). The benefit of knowledge-based diversity inputs could be enhanced on the condition that the reservoir of task-relevant knowledge and skills is shared and applied among team members to problem solving and is thus converted into social capital (Jia et al., 2014). Prior studies on the relationship between knowledge diversity and TC have omitted the idea-sharing process in the team. Further research is recommended to directly explore the idea-sharing intention of team members using a multilevel model (Park et al., 2018). Accordingly, this study attends to examine KS as a key mediating mechanism through which team diversity leads to TC.

Additionally, there is another research gap in the link between knowledge-based diversity and TC. Current studies exploring creativity have attended to either individual-level creativity [e.g., team member creativity (TMC)] or collective-level creativity (e.g., TC or organizational creativity) (Kim et al., 2019). However, collective-level creativity could not only be predicted by individual-level creativity but also be a mere result of individual efforts. Indeed, these two types of creativity at the measurement level depict different phenomena in a real organizational context. Thus, this study contends that it is both meaningful and necessary to decompose the roles of individual-level and collective-level creativity (e.g., TMC and TC) when exploring the diversity–creativity link in the team context. In this regard, the prior literature on the link between individual-level and collective-level creativity is deficient in both validating theoretical reasoning and rigorous empirical testing (Kim et al., 2019). As such, this study involves these two levels of creativity into an integrated model to examine the relationship between diversity and creativity.

Taken together, this study attempts to explore how knowledge-based diversity contributes to team-level creativity sequentially through the mediating mechanisms of KS and TMC. Relying on a group creativity model (Paulus and Dzindolet, 2008), group structure as group input could substantially impact individual cognitive processes and social contagion processes and thus eventually foster group output in the form of group creativity. Given that team diversity functions as group input, knowledge-based diversity could enhance individual cognitive processes (e.g., KS) and social contagious processes (e.g., individual-level creativity to team-level creativity; Burt, 1987; Ryan et al., 1996; Chen et al., 2013) and thus foster team-level creativity.

Notwithstanding the significance of exploring the effect of knowledge-based diversity on collective-level creativity (e.g., team-level creativity), few current studies have applied a multilevel approach to take the levels at which model constructs are measured into account. Specifically, although various mediating mechanisms have been involved in exploring the diversity–creativity link by prior studies, there are some flaws in that these studies have been mostly conducted only at the single-level or same-level analysis and assessment [e.g., educational specialization heterogeneity → TC at the team level, Shin and Zhou (2007); team knowledge diversity → TC at the team level, Park et al. (2018); team diversity → creativity of work teams at the team level, Sung and Choi (2019); or educational level diversity → TC at the team level, Guo et al. (2021)]. In this respect, a small body of prior studies have applied the multilevel approach to analyze the effect of team-level knowledge-based diversity on individual-level KS and TMC, which in turn fosters team-level creativity. Therefore, given that entrepreneurial team knowledge diversity (ETKD) and TC are organizational phenomena found at the collective level, KS and TMC are members' perceptions and behaviors found at the individual level. In contrast to single- or same-level analyses, a multilevel model analysis is an appropriate approach for examining individual-level mediating mechanisms through which team-level ETKD affects team-level creativity.

In sum, there is a black box present in the prior empirical research concerning team diversity; specifically, the underlying mechanism through which knowledge-based diversity leads to organizational outcomes, especially TC, is rarely tested. To further advance the literature concerning TC, this study aims to open this black box by exploring and testing a sequential intermediating mechanism determined by both KS and TMC at the individual level, through which team-level ETKD contributes to team-level creativity. To this end, based on a data set consisting of 252 team members nested with 42 entrepreneurial teams in China, multilevel structural equation modeling (MSEM) is utilized to examine the top-down relationship between ETKD and KS, as well as the bottom-up link between TMC and TC.

**Figure 1:**
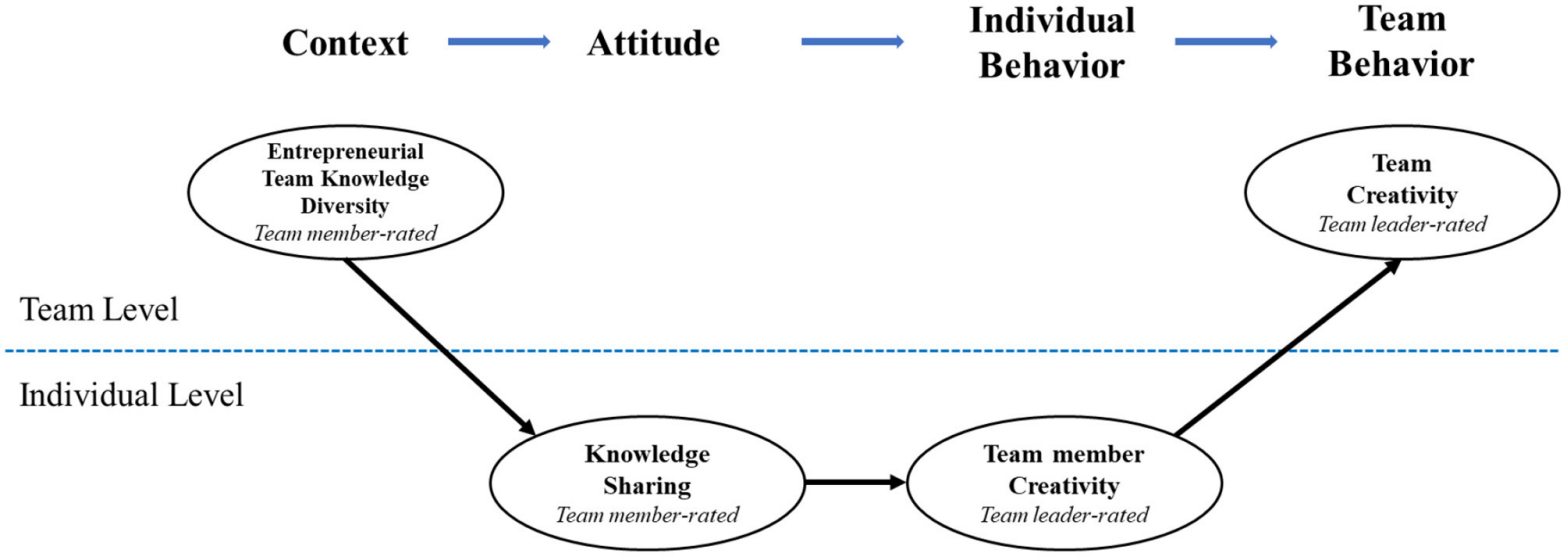
Hypothesized model.

This study aims to make the following contributions to the literature in the domains of value-in-diversity, creativity, and entrepreneurial teams. First, our findings specify the antecedent of TC from the view of knowledge-based diversity to broaden the empirical scope and generalizability of the diversity–creativity literature. Second, this study points out that the benefits of knowledge-based diversity could be achieved via the mediating role of KS among team members, thus realizing the potential of TC on the basis of various resources. Therefore, it sheds new light on the diversity–process-outcome relationship by taking KS as an intervening mechanism. Third, this study differentiates the role of individual-level and team-level creativity and integrates them into a multilevel model when exploring the relationship between ETKD and TC to enrich the creativity literature. Fourth, our findings indicate that knowledge-based diversity could engender collective-level team benefits through the individual-level sequential mechanism of KS and TMC. Our research is among the first to theoretically model and empirically test the multilevel mediation model of knowledge-based diversity in teams, particularly examining both top-down (e.g., ETKD to KS) and bottom-up (e.g., TMC to TC) relationships and thus bridging micro and macro domains, which is arguably one of the current challenges in the management literature (Aguinis et al., 2011; Mathieu and Chen, 2011). Finally, entrepreneurial teams have been under-researched despite the importance and significance of diversity and creativity in entrepreneurial teams. Our theoretical model is presented in Figure 1.

## Theoretical Orientations

### Entrepreneurial Team Knowledge Diversity and Team Creativity

What exactly is meant by an “entrepreneurial team” has induced a considerable debate among the academic community. According to Kamm et al. (1990), entrepreneurial teams are defined as “two or more individuals who jointly establish a firm in which they have a financial interest.” Furthermore, this definition has been broadened to involve individuals who have a direct influence on entrepreneurial firms' strategic choice (Gartner et al., 1994). Accordingly, Ucbasaran et al. (2003) combine both delineations by stating that the equity stake condition should be made stricter and that an individual could be regarded as a member of the entrepreneurial team on the condition that he or she has imposed a minimum equity stake.

Prior studies have thus far offered evidence that TMT heterogeneity in terms of demographic characteristics can contribute to explaining organizational outcomes (e.g., Carpenter, 2002). Most studies take the educational and functional heterogeneity of a team as a proxy for cognitive diversity when exploring the relationship between team heterogeneity and organizational outcomes. The findings of such studies indicate that due to diversity of experience and competencies, heterogeneity teams can produce more diverse ideas than can homogenous teams, which leads to a higher level of creativity (Flatt, 2004). According to Bantel and Jackson's (1989) study, heterogeneous TMTs in established firms in terms of team educational background are conducive to more substantial organizational innovation.

Following Madrid et al. (2016), an input–process–output (I–P–O) framework was employed to explore the creativity of groups or teams. In light of the I–P–O perspective, the diversity of teams in terms of task-related resources within a given team can improve the team's creativity potential. In this sense, TC will be higher when team members have diverse and non-redundant task-related resources, while TC will not differ from team-member individual creativity when the task-related resources of team members in a team are homogeneous and thus redundant.

According to Zhang et al. (2007), the knowledge diversity of a given team is conceptualized as the degree to which the reservoir of task-relevant knowledge and skills is specialized and distributed among team members. Specialized technical knowledge and skills in the particular domain of the tasks, as well as cognitive style and knowledge of creative heuristics, are considered to be important for coming up with creative ideas (Amabile, 1983). Given that creativity-related activities involve producing new things by combining previously unrelated processes, products, and materials, borrowing ideas, insights, or practices from one domain and modifying them for another domain or context (Amabile, 1996), TC could potentially be enhanced by cognitive resources resulting from the knowledge-based diversity of teams (Shin and Zhou, 2007).

When translated to entrepreneurial teams, these findings suggest that the team's reservoir of task-relevant knowledge should be diverse and, as result, help to generate creativity. In this vein, for venture teams under the assigned task domain, the educational or occupational backgrounds of each team member may not necessarily be associated with the task domain when pursuing TC. Accordingly, this study points out that compared with entrepreneurial teams with homogenous task-related knowledge among team members, entrepreneurial teams with heterogeneous reservoirs of knowledge related to the task domain will have higher creativity performance.

### Entrepreneurial Team Knowledge Diversity and Knowledge Sharing

According to Joshi and Roh (2009), when considering knowledge diversity in terms of task-related or work-related attributes (e.g., educational specialization, work experience, functional background), professional diversity could generate positive performance from the perspective of information and decision making. Salient social categories and a discriminatory climate are less prone to be instigated by the team's knowledge diversity, which is based on non-demographic attributes (e.g., educational specialization and functional background). In contrast, informational diversity can spur team members to share their unique views and distinct experiences and pool their various cognitive resources to find better and new solutions. In this sense, professionalism might be stimulated by these organizationally bound attributes (Perry-Smith and Shalley, 2003; Kearney and Gebert, 2009). This type of information-related or knowledge-based diversity could expand skills and perspectives for team members; thus, they can pool their diverse cognitive resources through increased levels of KS to achieve better decisions (Harrison and Klein, 2007).

Hoever et al. (2012) noted that team members can be stimulated to find unique combinations and recombinations of existing ideas by knowledge-based diversity that offers a reservoir of knowledge and non-redundant information. In this vein, when translated to entrepreneurial teams, the benefits of team diversity can be realized to the extent that team members with various backgrounds fully exploit diversity through the free flow of diverse knowledge, information, and ideas, thus boosting the encounter and interaction of various perspectives among members (Jia et al., 2014; Sung and Choi, 2019).

Accordingly, the following hypothesis is proposed:

Hypothesis 1. Team-level entrepreneurial team knowledge diversity is positively associated with individual-level knowledge sharing.

### Knowledge Sharing and Team Member Creativity

Knowledge Sharing is defined as the individual-level intention to exchange and expose information and ideas among team members. Leenders et al. (2003) noted that the presence of diverse intragroup cognitive resources offers the raw materials necessary for creativity potential. Sung and Choi (2019) further contended that the presence of only a knowledge pool within the group or team fails to lead to creativity, which occurs only when members are willing to share and apply the knowledge reservoir. Knowledge Sharing enables members to have access to diverse knowledge resources within the group, which results in interacting and co-learning among members, thus promoting the utilization of intragroup cognitive resources to creatively solve problems (Leenders et al., 2003). Furthermore, in line with the social structural perspective, the coordinated action, and the development of new ideas can be fostered by the intense activities of knowledge-sharing and dense networks of communication (Perry-Smith and Shalley, 2003; Jia et al., 2014).

According to Chae et al. (2015), individual creativity is conceptualized as an individual capability to generate new and novel ideas. Amabile (1996) noted that there are three types of determinants of individual creativity: domain-related skills, creativity-related skills, and task motivation. Thus, KS is considered a catalyst for creativity-related collaboration in that knowledge is one of most essential and important domain-related skills. In this sense, KS represents a process of communication within the team in which members might rebuild their knowledge when they access knowledge from other team members (Hendriks, 1999). During this process of KS and exchange, team members could also enhance their accumulation of knowledge and experience (Zhang et al., 2018). In line with Sternberg and Lubart's (1995) view, the knowledge accumulation of individuals can potentially inspire their creativity. Moreover, previous empirical studies also provide evidence for the relationship between KS and individual creativity. For instance, individual creativity is empirically verified to be enhanced by employee relationships, KS, and IT application maturity (Peng et al., 2014). On the basis of the above reasoning, when translated to entrepreneurial teams, this study points out that team members' perception of KS leads to their individual-level creativity.

Accordingly, the following hypothesis is proposed:

Hypothesis 2. Individual-level knowledge sharing is positively associated with individual-level TMC.

### Bottom-Up Influence of Team Member Creativity on Team Creativity

Prior studies indicate that individual-level creativity (e.g., TMC) could lead to team-level creativity and thus result in an upward effect on TC (Pirola-Merlo and Mann, 2004; Chen et al., 2013). Drawing from social contagion theory, behavior contagion could take place among team members by imitating other members' behaviors (Burt, 1987; Ryan et al., 1996). In this sense, through the social contagion process, individual-level phenomena such as creativity and innovative behavior foster related team-level phenomena (Burt, 1987; Ryan et al., 1996; Chen and Kanfer, 2006; Chen et al., 2013). Specifically, when a given member with a high level of creativity attempts to yield novel task outcomes by integrating and coordinating the inputs of other members, this member's behavior might emerge as behavior norms that other team members would be likely to follow. As such, the creative behavior among the members would spread contagiously throughout the team and thus be collectively formed at the team level, thereby resulting in collective-level outcomes.

Kozlowski and Klein (2000) noted that the upward contribution by members to TC is a micro-to-macro, cross-level organizational phenomena per se. However, prior studies concerning the effect of individual-level creativity have mostly distinguished average individual creativity from TC and have been widely conducted at the team level (Pirola-Merlo and Mann, 2004; Chen et al., 2013; Kim et al., 2019). Other than the lack of theoretical explanation for the cross-level bottom-up effect, the empirical research methodology has also been limited to single-level or same-level analyses.

According to Croon and van Veldhoven (2007), inappropriate estimates of the standard errors of the regression parameters could be generated in that the variability of the survey data might be reduced when individual-level variables are aggregated to the team-level variables. Thus, this study proposes a multilevel relationship between individual-level creativity (e.g., TMC) and team-level creativity (e.g., TC) with the aim of mitigating this theoretical and empirical gap.

Accordingly, the following hypothesis is proposed:

Hypothesis 3. Individual-level team member creativity is positively associated with team-level creativity.

### Multilevel Mediation Through Knowledge Sharing and Team Member Creativity

Encountering mixed and inconclusive empirical findings, diversity researchers suggested that to examine the relationship between the knowledge-based heterogeneity of teams and the related organizational outcomes, the exclusive focus on the direct effects of team diversity has been limited and insufficient in interpreting the underlying mechanism due to omitting the intervening variables, such as team decision-making, team conflict, team communication (Henneke and Lüthje, 2007). In this regard, however, the diverse literature has omitted knowledge-related processes, because previous prevailing research concern has mostly with interpersonal challenges, such as stereotyping, dissent, and conflict (van der Vegt, 2002; Cunningham and Sagas, 2004), which have been considered of limited value-adding importance when investigating positive team outcomes, particularly creativity (van Knippenberg et al., 2004). Given that knowledge-sharing and knowledge-exchange are core catalysts of TC potential (Taylor and Greve, 2006; Zhou et al., 2009; Sung and Choi, 2019), this study denotes that KS among team members could be identified as a critical intervening process to further explore the relationship between diversity and creativity.

Rather than separating information/decision-making theory and social categorization theory, the categorization-elaboration model integrates these two perspectives by identifying the underlying mechanisms of the effects of team diversity (Sung and Choi, 2019). The categorization–elaboration model points out that team diversity can have positive effects on team outcomes (e.g., decision quality and creativity) in that the elaboration of task-related information among members is elicited by diversity, thereby underscoring the importance and significance of the intervening process, which can explain the diversity–performance link (van Knippenberg and Schippers, 2007). According to van Knippenberg et al. (2004), the concept of elaboration is defined as a multilevel, multifaceted construct involving diverse cognitive and interactive processes that can take place at both the individual and group levels of analysis. In light of this model, prior empirical research provides evidence for the mediating role played by information elaboration in the diversity–performance link (Kearney and Gebert, 2009; Pieterse et al., 2013). This study extends this reasoning by identifying KS as an individual-level, intervening process that mediates the relationship between team diversity and TC.

Furthermore, prior studies have noted that the relationship between team diversity and KS can foster the team's capability to generate creative ideas (Taylor and Greve, 2006; Zhou et al., 2009). In this sense, KS provides a venue through which team members can access and accumulate diverse cognitive resources, and this flow of knowledge or informational resources fertilizes TC potential (Sung and Choi, 2019). Team members transform cognitive resources stemming from members of diverse backgrounds into actual creative performance through the process of KS, and thus, the social capital of the team is generated (Hoever et al., 2012; Han et al., 2014). Based on the above reasoning, when translated to entrepreneurial teams, knowledge-based or informational diversity is apt to stimulate task orientation to share distinct information and diverse experiences, thereby facilitating interactions among team members. Therefore, this study predicts that KS offers a conduit through which the effects of knowledge-based diversity are transmitted to TC potential.

Integrating our theoretical development for Hypotheses 2 and 3 with the above arguments, this study points out that individual-level KS and TMC sequentially mediate the relationship between team-level knowledge-based diversity and team-level creativity. Grounded in a group creativity model (Paulus and Dzindolet, 2008), important inputs functioning by group, task, and situational variables (e.g., group member characteristics, group structure, group climate, and external demands) could stimulate operating processes (e.g., cognitive, motivational, and social processes), after which the operating processes could generate group-level creativity-related outputs. In this vein, knowledge-based diversity as a group structure could foster group creativity in a given organization (e.g., TC) by influencing individual cognitive processes (e.g., KS and individual creativity) and social processes (e.g., social contagion). Relying on the social contagious process, individual creativity proliferates among other team members and eventually enhances TC. Taken together, when translated to entrepreneurial teams, knowledge-based diversity could foster TC sequentially by influencing KS (i.e., the proximal mediator) and TMC (i.e., the distal mediator).

Accordingly, the following hypothesis is proposed:

Hypothesis 4. Team-level entrepreneurial team knowledge diversity will significantly and indirectly relate to team-level creativity sequentially through individual-level knowledge sharing and team member creativity.

## Research Design and Methods

### Sample and Procedures

Entrepreneurial teams that were concerned with the importance of creative capability (e.g., telecommunication, electronics, bioengineering, informational technology, service consultation) were priority research samples in that these kinds of entrepreneurial teams are relatively more sensitive to creativity than are entrepreneurial teams in other industries.

The Pearl River Delta region of Guangdong province is one of the most developed regions in China and is a leading region in the process of China's reform and opening. Nowadays, the Pearl River Delta region fosters a strong entrepreneurial and creative atmosphere and generates a large number of entrepreneurial practices and creative activities. Specifically, our research team distributed questionnaires to members of entrepreneurial teams from Entrepreneurial and Creative Parks (such as Southern Software Park, Jinjia Creative Valley, V12 Pioneer Park, etc.) in the Pearl River Delta of China, mainly in the cities of Guangzhou, Shenzhen, and Zhuhai. Following Ucbasaran et al.'s (2003) recommendation for the equity stake condition, individuals in the survey teams have imposed a minimum equity stake; thus, they can be considered as members of the entrepreneurial team. Finally, the survey data were collected from 252 team members nested under 42 entrepreneurial teams.

Our research team directly contacted the directors of entrepreneurial and creative parks, and the entrepreneurial teams in these parks were randomly selected. A total of 275 team members from 42 entrepreneurial teams completed the survey, and a dyad data set of 252 member-leader matched data sets comprised our final sample, indicating a response rate of 91.6%.

In the survey process, participants were informed about the managerial implications of this research and the importance of careful observation of survey items, and two different types of questionnaires including team member surveys and leader surveys were offered. In the case of the team member survey, ETKD and KS were measured, and the sample consisted of 61% men and 39% women. The age groups were as follows: 5.9% of the respondents were in their 20s, 58.0% were in their 30s, 18.6% were in their 40s, 15.4% were in their 50s, and 2.1% were in their 60s. Regarding education level, 15.4% of the respondents had high school education, 29.8% had a college education, 42.4% had a bachelor's degree, 10.7% had a master's degree, and 1.7% had a doctoral degree. In the case of the team leader survey, the levels of EC and TC were rated. The leader demographics consisted of 89% males and 11% females. The entrepreneurial team size ranged from 5 to 10 members, with an average size of six members.

Due to the different sources of survey data, including team member surveys and leader surveys, the issue of a potential common method basis was minimized. In addition, common method bias was checked by Harman's one-factor test. By the principal component factor method, three items of ETKD, five items of KS, four items of TMC, and the six items measuring TC were entered into the analysis. The results showed that 37.71% of the variance was explained by the first factors in the model. Thus, common method bias was not an issue.

### Measures

All measures in this study were translated into Chinese. Following Brislin's (1980) translation-back-translation procedure, the translation from English to Chinese and then back to English was performed by bilingual experts with the aim of verifying the translation. Appendix lists the questionnaire of key measurements in this study.

### Entrepreneurial Team Knowledge Diversity

Entrepreneurial team knowledge diversity was measured with the scale adopted from Campion et al. (1993) and Jehn et al. (1999). Team members were asked to respond to how extensively they perceive the knowledge diversity of their team to be. The three items of the scale measure were rated on a Likert-type scale ranging from 1 (I do not agree at all) to 7 (I completely agree). Sample items were “Your entrepreneurial team members vary widely in their professional fields” and “Your entrepreneurial team members have a variety of different educational backgrounds and experiences.” Considering that ETKD was conceptualized at the team-level construct, the individual-level data were aggregated to the team-level. According to LeBreton and Senter's (2008) aggregation procedure, based on a multilevel random-intercept model, the results showed that the mean R_wg(j)_ of ETKD was 0.73 (SD = 0.35), i.e., greater than the recommended value of 0.70. Entrepreneurial team knowledge diversity modeled as a group-level construct can be formulated by aggregating individual-level ETKD ratings. Based on one-way ANOVA, the results showed that ETKD has a high between-level variation and within-level agreement [*F* = 1.614, *p* < 0.05; ICC1 (intraclass correlations) = 0.32; ICC2 (reliabilities of the group means) = 0.83; Bliese (2000)]. Moreover, multilevel confirmatory factor analysis (MCFA) can prevent conflation in reliability estimates at the within level and between level because the measurement model parameters are decomposed into level-specific parts (Geldhof et al., 2014). In this sense, the MCFA approach can be applied to evaluate the Cronbach's alpha within and between levels of the model constructs (Koopmann et al., 2016). The MCFA analysis results indicated that the Cronbach's alpha at the between-group level of ETKD was 0.971, while the Cronbach's alpha at the within-team level of ETKD was 0.713, thereby demonstrating that the measure was reliable at both levels.

### Knowledge Sharing

Knowledge sharing was measured with the scale adopted from Bock et al. (2005), including explicit KS and implicit KS. Team members were asked to rate how extensive they felt their intention toward KS was. The five items of the scale measure were rated on a Likert-type scale ranging from 1 (I do not agree at all) to 7 (I completely agree). A sample measured item of explicit KS was “I will always provide my manuals, methodologies and models for members of my team.” A sample item measuring tacit KS was “I will try to share my expertise from my education or training with other team members in a more effective way.” Notably, the validity and reliability evidence for the scale have been demonstrated by prior studies. Based on one-way ANOVA, the results showed that KS had a high between-level variation and within-level agreement (*F* = 2.029, *p* < 0.05; ICC1 =0.38; ICC2 =0.75). Moreover, the measure was reliable at both between and within levels, as indicated by Cronbach's alpha based on MCFA (Geldhof et al., 2014), which was 0.865 at the between-team level and 0.701 at the within-team level.

### Team Member Creativity

Team member creativity was measured with the scale developed by Tierney and Farmer (2011). Team leaders were asked to rate how extensive they felt their team members' individual creativity was. The four items of the scale measure were rated on a Likert-type scale ranging from 1 (I do not agree at all) to 7 (I completely agree). Sample items were “This team member seeks new ideas and ways to solve problems” and “This team member identifies opportunities for new ways of dealing with work.” Notably, the validity and reliability evidence for the scale have been demonstrated by prior studies. Based on one-way ANOVA, the results showed that EC had a high between-level variation and within-level agreement (*F* = 1.705, *p* < 0.05; ICC1 = 0.35; ICC2 = 0.83). Moreover, the measure was reliable at both between and within levels, as indicated by Cronbach's alpha based on MCFA (Geldhof et al., 2014), which was 0.980 at the between-team level and 0.945 at the within-team level.

### Team Creativity

Team creativity was measured with the scale developed by George and Zhou (2001). Following prior research suggestions (Pirola-Merlo and Mann, 2004), team leaders can be reliable sources of team-level information; thus, team leaders were asked to rate how extensive they felt their TC was. The six items of the scale measure were rated on a Likert-type scale ranging from 1 (I do not agree at all) to 7 (I completely agree). Sample items were “Your team often comes up with new and practical ideas to improve performance” and “Your team suggests new ways to achieve goals or objectives.” Notably, the validity and reliability evidence for the scale have been demonstrated by prior studies. The team-level Cronbach's alpha for this scale was 0.963.

### Controls

Several control variables were considered to bias our research. First, team members' gender and age were taken as control variables because prior studies have demonstrated that gender and age are associated with creativity (e.g., Gong et al., 2009; Richter et al., 2012). Second, team members' educational level was also included as a control variable because the educational level of team members might influence their creativity (Amabile, 1996; Shin and Zhou, 2003; Shin et al., 2012). Third, following the recommendation of Koopmann et al. (2016), team tenure and team size were assessed as control variables at the team level.

### Analytical Strategy

Considering the nested nature of our research data (i.e., team members were nested within their leaders), according to Preacher et al. (2010), MSEM was applied to examine the multilevel model of this study, in which it is necessary to model both top-down and bottom-up relationships.

In comparison with hierarchical linear modeling (HLM), MSEM can not only evaluate indirect relationships in the multilevel model more precisely by means of decomposing variance into components at the between- and within-levels because potential problems of conflated within- and between-level relationships are avoided (Zhang et al., 2009; Preacher et al., 2010) but can also test upward impacts in the multilevel model, which fails to be assessed by traditional multilevel modeling techniques (e.g., HLM; Preacher et al., 2010).

The indirect relationship model was suggested by Hypotheses 7–9 of this study, in which ETKD and TC were sequentially mediated by KS and TMC. By applying MSEM, this study could evaluate the top-down link between ETKD and KS, the relationship between KS and TMC at the individual level, and the bottom-up link between TMC and TC simultaneously. Furthermore, the indirect effects between ETKD and TC were mediated, although two individual-level mediators (KS and TMC) were quantified by the product-of-coefficients method.

In particular, to test the proposed model's top-down link (i.e., 2–1 relationship), following Preacher et al. (2010) and Zhang et al. (2009), the structural coefficient of the relationship between the level-2 predictor (ETKD) and the latent group mean of the level-1 outcome (KS) was examined. To test the proposed model's bottom-up link (i.e., 1–2 relationship), considering Lüdtke et al.'s (2008) approach to MSEM, which is an efficient method of testing the bottom-up relationship by a one-step, full information maximum likelihood estimation, the structural coefficient of the relationship between the latent group mean of TMC and the lant level-2 outcome (TC) was examined to assess the bottom-up link.

To test the multilevel mediation relationship (i.e., 2-1-2, 2-1-1-2), following the recommendations of Zhang et al. (2009), the chain links among the latent variables and latent group means at the between-group level were examined. In this sense, the mediation effect and chain mediation effect were examined by multiplying the path coefficients among the latent predictor (ETKD), the latent group means of the mediators (KS and TMC), and the latent outcome (TC). Based on unstandardized coefficients of model paths, the point estimates and standard errors were obtained from the analysis for the multilevel mediation effects. To further test cross-level mediation effects, a parametric bootstrapping procedure was applied in terms of the recommendation by Preacher et al. (2010). A Monte Carlo simulation with 20,000 replications was conducted to test the 95% bias-corrected confidence interval (CI) around the indirect effects (e.g., Wang et al., 2013; Lanaj et al., 2014; Lennard et al., 2019).

In this study, all the analyses were implemented by using Mplus 8.0 (Muthén and Muthén, 1998–2010) with robust maximum likelihood (MLR) estimation. Based on Hu and Bentler's (1999) recommendations, means of the root-mean-square error of approximation (RMSEA), the Tucker–Lewis Index (TLI), and the comparative fit index (CFI) were employed to assess model fit, and chi-square difference testing was applied to compare multilevel alternative rival models.

## Results

### Descriptive Statistics

The summary statistics and correlations of all the model constructs are shown in Table 1. Notably, educational level as a control variable was unassociated with substantive model variables. Becker (2005) contended that, with the aim of avoiding reduced statistical power and increased type II errors, control variables that are uncorrelated with the dependent variable should be excluded from the model. In this case, following Becker's (2005) recommendations, the educational level variable was dropped from subsequent analysis.

**Table 1:** Means, standard deviations, and inter-correlations of variables.

**Variable**	**M**	**SD**	**1**	**2**	**3**	**4**	**5**
**INDIVIDUAL LEVEL**
Gender	1.60	0.49	1				
Age	2.54	0.92	0.198^*^	1			
Education level	2.54	0.94	0.072	−0.120	1		
Knowledge sharing	5.19	1.20	0.215^**^	0.121	−0.075	1	
Team member creativity	5.24	1.38	0.169^*^	0.208^**^	−0.016	0.588^**^	1
**TEAM LEVEL**
Team tenure	2.03	0.96	1				
Team size	6.52	3.18	−0.071	1			
Entrepreneurial team knowledge diversity	5.28	0.74	0.166^*^	0.213^**^	1		
Team creativity	5.46	0.73	0.196^*^	0.209^**^	0.577^**^	1	

*Individual level N = 252; team level N = 42. Gender was dummy-coded (0 = female, 1= male). Age was categorically measured (1 = 20–30 years; 2 = 31–40 years; 3 = 41–50 years; 4 = 51–60 years; 5 = ≥61). Education level was categorically measured (1 = high school, 2 = college, 3 = bachelor degree, 4 = master degree, 5 = doctoral degree). Team tenure was categorically measured (<1 year as 1, 1–4 years as 2, 4–7 years as 3, 7–10 years as 4, more than 10 years as 5)*.

* *p <0.05*.

** *p <0.01*.

### Convergent and Discriminant Validity

To guarantee the constructs' validity and reliability, confirmatory factor analysis (CFA) was conducted to evaluate the reliability, convergent validity, and discriminant validity of the measurement scales (see Table 2).

**Table 2:** Overall reliability of the constructs and factor loadings of indicators.

**Construct (source)**	**Items**	**Factor loading**	**SMC**	**Cronbach' alpha**	**CR**	**AVE**
Entrepreneurial team knowledge diversity	ETKD1	0.868	0.753	0.762	0.869	0.689
(Campion et al., 1993; Jehn et al., 1999)	ETKD2	0.817	0.667			
	ETKD3	0.803	0.645			
Knowledge sharing	KS1	0.839	0.704	0.808	0.880	0.598
(Bock et al., 2005)	KS2	0.817	0.667			
	KS3	0.789	0.623			
	KS4	0.780	0.608			
	KS5	0.622	0.387			
Team member creativity	TMC1	0.955	0.912	0.945	0.961	0.859
(Tierney and Farmer, 2011)	TMC2	0.943	0.889			
	TMC3	0.936	0.876			
	TMC4	0.872	0.760			
Team creativity	TC1	0.888	0.789	0.890	0.918	0.653
(George and Zhou, 2001)	TC2	0.883	0.780			
	TC3	0.855	0.731			
	TC4	0.837	0.701			
	TC5	0.721	0.520			
	TC6	0.631	0.398			

*SMC, square multiple correlation; CR, composite reliability; AVE, average variance extracted*.

For Cronbach's alphas and composite reliability coefficients, the model constructs were all found to be beyond the recommended threshold of 0.7, which demonstrated a high level of internal consistency. Regarding the factor loadings of the model constructs, all the factors were loaded significantly by their corresponding items and met the recommended cutoff of 0.7, thereby indicating an acceptable level of convergent validity; in terms of the square multiple correlation (SMC) values of model constructs, the results were all beyond the recommended minimum of 0.5, which showed acceptable item reliability. In addition, the average variance extracted (AVE) was beyond the recommended value of 0.5, which revealed that the amount of variance due to constructs' items was more than the amount of variance due to measuring error (Fornell and Larcker, 1981). Thus, according to the above gauging criteria, the convergent validity of the model constructs was guaranteed.

Following Fornell and Larcker (1981), to assess the discriminant validity of the model constructs, it was recommended to compare the square roots of the AVE values with the interconstruct correlations. As shown in Table 1, the square roots of the AVE values of all the model constructs were in all cases above the interconstruct correlation coefficients, which implies that the model constructs are presumed to possess discriminant validity.

Moreover, a series of CFAs was conducted to determine the distinctiveness of the model constructs. Specifically, the four-factor model was compared with seven alternative models, including four three-factor models, two two-factor, and one one-factor models. Considering the correlation among constructs, the first two-factor model was obtained by blending ETKD, KS, and TMC, and the second two-factor model was obtained by blending KS, TMC, and TC. In terms of both correlation among constructs and leader-rated or member-rated variables, four three-factor models were constituted. The one-factor model was obtained by combining all four constructs into one latent factor. Based on chi-square statistics and the fit indices of CFI, TLI, RMSEA, and SRMR, the distinctiveness of the models was assessed to test whether the proposed measurement model adequately fit the data. The CFA results showed that the proposed four-factor model fits the data better than other alternative nested models (see Table 3), thereby indicating that our measurement models captured the distinctiveness of research constructs and confirmed our modeling approach.

**Table 3:** Results of confirmatory factor analysis.

**CFA model**	**χ^**2**^**	***df***	**CFI**	**TLI**	**RMSEA**	**SRMR**
**One factor model**	663.68	135	0.749	0.715	0.155	0.077
*ETKD, KS, TMC, and TC were blended*						
**Two factor model**	507.28	134	0.823	0.797	0.131	0.101
*ETKD, KS, and TMC were blended*						
**Two factor model**	607.63	134	0.775	0.743	0.148	0.073
*KS, TMC, and TC were blended*						
**Three factor model**	298.62	132	0.921	0.908	0.088	0.055
*ETKD and TC were blended*						
**Three factor model**	303.23	132	0.919	0.906	0.089	0.055
*ETKD and KS were blended*						
**Three factor model**	415.72	132	0.865	0.844	0.115	0.096
*KS and TMC were blended*						
**Three factor model**	545.60	132	0.803	0.772	0.139	0.071
*TMC and TC were blended*						
**Four factor model**	242.51	129	0.946	0.936	0.074	0.049

*χ^2^, chi-square value; df, degree of freedom; CFI, confirmatory fit indices; TLI, Tucker-Lewis index; RMSEA, root mean square error of approximation; SRMR, standardized root mean square residual. ETKD, entrepreneurial team knowledge diversity; KS, knowledge sharing; TMC, team member creativity; TC, team creativity*.

### Hypothesis Testing

Based on MSEM, the proposed multilevel structural model showed a good fit (χ^2^ = 347.71, df = 189, CFI = 0.92, TLI = 0.91, RMSEA = 0.07), in which ETKD and TC are associated through KS and TMC.

In the structural model, the proposed direct and indirect relationships were tested (shown in Table 4). On the one hand, for all direct relationships, first, ETKD was positively related to KS, as the results showed a significant unstandardized path coefficient (β = 0.757, *p* < 0.001), thus supporting Hypothesis 1. Second, ETKD was not positively associated with EC or TC, as the results indicated non-significant unstandardized path coefficients (β = 0.146, *p* > 0.05; β = 0.188, *p* > 0.05). Third, KS was significantly associated with TMC, as indicated by a significant unstandardized path coefficient (β = 0.683, *p* < 0.001), thus supporting Hypothesis 2. Fourth, TMC was significantly associated with TC, and KS was also positively related to TC, as shown by significant unstandardized path coefficients (β = 0.307, *p* < 0.05; β=0.503, *p* < 0.01), thus supporting Hypothesis 3.

**Table 4:** Tests of direct and indirect relationships (Hypotheses 1–4).

**Path**	**Estimates**	**s.e**.	**Lower and upper 95% CI limits**
**Test of direct relationships**			
Top-down direct path (2–1)			
Entrepreneurial team knowledge diversity → knowledge sharing	0.757^***^	0.177	[0.410, 1.103]
Entrepreneurial team knowledge diversity → team member creativity	0.146	0.203	[−0.252, 0.544]
Entrepreneurial team knowledge diversity → team creativity	0.188	0.109	[−0.026, 0.402]
Direct path (1–1)			
Knowledge sharing → team member creativity	0.683^***^	0.147	[0.395, 0.971]
Bottom-up direct path (1–2)			
Team member creativity → team creativity	0.307^*^	0.123	[0.066, 0.547]
Knowledge sharing → team creativity	0.503^**^	0.147	[0.215, 0.791]
**Test of indirect relationships**			
Indirect paths model			
Entrepreneurial team knowledge diversity → knowledge sharing → team creativity	0.381^*^	0.177	[0.034, 0.727]
Indirect paths model			
Entrepreneurial team knowledge diversity → team member creativity → team creativity	0.045	0.074	[−0.100, 0.190]
Complete indirect paths model (2–1–1–2)			
Entrepreneurial team knowledge diversity → knowledge sharing → team member creativity → team creativity	0.159^**^	0.051	[0.059, 0.258]

*For direct relationships (upper panel) and indirect relationships (lower panel), unstandardized estimates are reported. 1, level-1 variable; 2, level-2 variable; CI, confidence interval. Significant direct and indirect effects using Monte Carlo confidence intervals*.

* *p <0.05*.

** *p <0.01*.

*** *p <0.001*.

On the other hand, regarding indirect relationships, following recommendations by Preacher et al. (2010), the multilevel mediating effect of KS and TMC on the relationship between ETKD and TC (i.e., 2-1-1-2 model) was tested. In the proposed multilevel mediation model, path A (ETKD → KS), path B (KS → TMC), and path C (TMC → TC) were evaluated simultaneously. Preacher et al. (2010) noted that multilevel indirect effects are evaluated at the between-group level in situations in which the model involves both downward and upward effects. First, ETKD had a significant indirect relationship with TC through KS, as indicated both by a significant unstandardized estimate of the product of coefficients (γ = 0.381, *p* < 0.05), and the 95% bias-corrected CI around the indirect effect (CI = [0.034, 0.727]). Second, ETKD failed to have a significant indirect relationship with TC through TMC, as results indicated that unstandardized estimate of the product of coefficients was not significant (γ = 0.045, *p* > 0.05), and the 95% bias-corrected CI around the indirect effect included zero (CI = [−0.100, 0.190]). Third, ETKD had a significant indirect relationship with TC through the chain of KS and TMC, as indicated both by a significant unstandardized estimate of the product of coefficients (γ = 0.159, *p* < 0.01) and the 95% bias-corrected CI around the indirect effect (CI = [0.059, 0.258]); this result supports Hypothesis 4.

**Figure 2:**
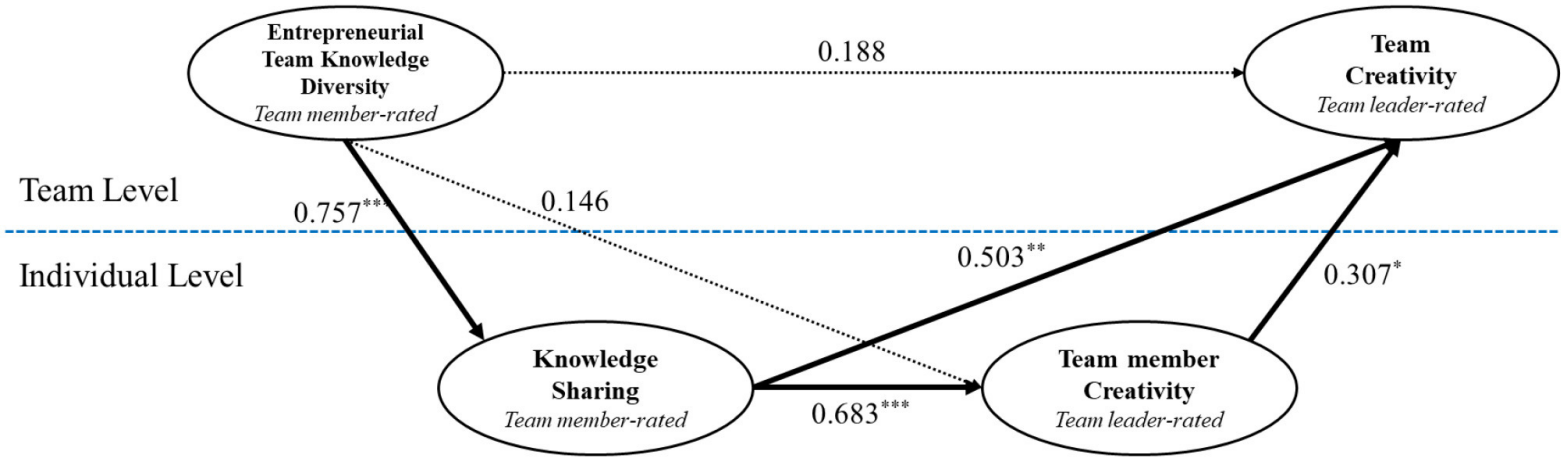
Results of multilevel sequential mediation analysis. *p < 0.05, **p < 0.01, ***p < 0.01; Unstandardized coefficients are presented.

To further test the full vs. the partial mediation of the proposed model, this study examined a rival model in which the direct path from ETKD to TC was fixed to zero. Following De De Wulf et al.'s (2001) recommendation, a comparison between the proposed model and the rival model was conducted in terms of model fit indices and the proportion of statistically significant paths. The results showed that the fit index values for the rival model (χ^2^ = 355.29, df = 188, CFI = 0.92, TLI = 0.90, RMSEA = 0.07) failed to improve the model fit. Furthermore, the scaled chi-square difference test indicated that the fit of the rival model was similar to that of the proposed model [$\Delta \chi_{\text{scaled}}^{2}$(1) = 0.35, *p* = n.s.]. Thus, the examination provided evidence for the full mediation of the proposed model. The results of multilevel sequential mediation analysis are shown in Figure 2.

## Discussion

With the aim of providing empirical evidence for the link between ETKD and TC, this study explores the underlying mechanism by which the knowledge composition of venture teams affects their creativity. Based on a data set of 252 team members from 42 entrepreneurial teams in China, MSEM is employed to assess the top-down relationship between ETKD and KS, as well as the bottom-up link between TMC and TC. The findings fail to support the research hypothesis of a direct effect of ETKD on TC. Indeed, the indirect effect has been verified; specifically, KS and TMC play mediating roles in this link. In the following, this study offers research and practical implications, as well as limitations that warrant future research.

### Research Implications

This study makes theoretical and empirical contributions to the literature concerning diversity, creativity, and entrepreneurial teams based on our findings.

First, this study concerns knowledge-based diversity as the main heterogeneity variable by which to explore the diversity–creativity relationship. According to Williams and O'Reilly's (1998) suggestion, the heterogeneity variable should be chosen properly in terms of its conceptual association with the outcomes of interest in that the effects of different heterogeneity variables are not all similar. In the case of the creativity of venture teams such as those in our sample, knowledge-based diversity offers various perspectives, knowledge, and skills that are beneficial for the creativity potential of teams. Thus, knowledge-based diversity is the most related heterogeneity variable. In line with the results of meta-analytic research by Joshi and Roh (2009), compared with demographic diversity (e.g., gender, age, and race), informational diversity (e.g., function, education, and tenure diversity) can significantly predict the positive performance of work units. Thus, potential informational benefits stemming from the non-redundant professional backgrounds of the constituting members could be engendered by knowledge-based diversity.

Second, this study further extends the literature on the diversity–creativity link by disclosing that individual-level KS, as a critical intervening process, plays a mediating role in the relationship between knowledge-based diversity and TC. Consistent with the input-process-output model of group effectiveness (Horwitz and Horwitz, 2007), exploring the implications of knowledge-based diversity in terms of intervening processes could answer the call to open the black box in the link between diversity and outcomes. Horwitz and Horwitz (2007) noted that a given group could have access to a broad range of cognitive knowledge and skills originating from the background diversity of constituting members. In this case, different roles are distributed naturally, and different aspects of problem solving are borne for constituting members with different professional backgrounds. As such, team members could readily derive, generate, and accept different perspectives on the basis of their diverse professional backgrounds (Bunderson and Sutcliffe, 2002). In this sense, on the one hand, unnecessary conflicts and competition among team members could be mitigated and softened by this functional diversity among members; on the other hand, positive and cooperative intrateam exchanges and subsequent creative outcomes could be yielded by the diversity in ideas and knowledge. Accordingly, attention to intermediate group processes should be paid as these processes may be proximal to the input of group composition and be relevant to output in question; as such, the diversity–creativity link could be more clearly clarified (van Knippenberg et al., 2004).

Third, this study differentiates the role of individual-level and team-level creativity and merges them into an integrated model when exploring the relationship between ETKD and TC. Considering that the two levels of creativity known to be highly related tend to describe different organizational phenomena, this study attempts to explore the diversity–creativity link by decomposing the role of the two types of creativity in a unified multilevel model and thus to enrich the creativity literature.

Fourth, this study examines the underlying mediation mechanism in the relationship between ETKD and TC. Relying on a group creativity model (Paulus and Dzindolet, 2008), knowledge-based diversity could enhance TC potential through the underlying sequential mechanism of individual-level KS and TMC. In light of a group creativity model, ETKD, which is considered a critical input, could promote the team creative process by initiating operating processes (e.g., cognitive, motivational, and social processes), such as team members' KS and individual creativity. Moreover, the enhanced operating processes could ultimately yield output from team-level creativity through social contagious processes. By examining KS and TMC as the mediating roles that provide a conduit to transmit the effect of knowledge diversity of venture teams to teams' creativity potential, a new process mechanism of TC has been revealed. Accordingly, this study contributes to extending the understanding of the nature of team processes for diversity–creativity links.

Moreover, given that prior studies on the diversity–creativity link have explored the underlying intervention mechanism under which team diversity influences TC mostly based on single-level or same-level analysis, this study contributes to the literature by methodological advancement. In this regard, when examining the effect of team-level diversity on individual-level team members' cognition and creativity and in turn diffusing among the team to foster team-level creativity, the current research pays less attention to multilevel or cross-level methods. Considering that team members' KS and creativity are individual-level phenomena and that team diversity and creativity are team-level organizational phenomena, the single-level or same-level method fails to fully depict and interpret the intrateam interactions. Therefore, this is why the MSEM approach is applied to assess the proposed multilevel mediation model, which consists of both top-down relationships (level 2 → level 1) and bottom-up relationships (level 1 → level 2), and thus contributes to understanding the dynamic relationship between individual members in the team and the team as whole.

Finally, our study explores the diversity–creativity link in entrepreneurial teams with a field nested dataset from venture teams, especially in the domain of high-tech industries in China. Thus, our findings provide some of the first empirical evidence to examine how knowledge-based diversity of entrepreneurial teams facilitates TC potential, given that the prior studies on diversity–creativity relationships have been most concerned with working teams, R&D teams, and TMTs (e.g., Shin and Zhou, 2007; Buyl et al., 2011; Wang et al., 2016; Park et al., 2018; Sung and Choi, 2019; Guo et al., 2021).

### Practical Implications

According to our multimodel test, the results indicate that ETKD can lead to enhanced TC. Most new venture teams were found to be constructed based on personal and professional relationships and thus to be more homogeneous with regard to team members' knowledge backgrounds (Henneke and Lüthje, 2007). In this case, major implications concerning the prefounding and postfounding stages of entrepreneurial teams should be considered for entrepreneurial practitioners.

First, for the prefounding stage of new entrepreneurial teams, it is recommended to underline the importance of fostering teams with heterogeneous compositions in terms of knowledge. The formation and evolution of new venture teams is often a random process mostly on the basis of previous private and professional relationships. Thus, entrepreneurial practitioners are necessary to intervene in the prefounding stage to pay attention more carefully to its composition and more consciously foster entrepreneurial teams.

In this regard, it is necessary for entrepreneurship educators, research institutions, and venture capitalists to enhance the intention of a leading entrepreneur or technical-oriented core venture team to take in team members with diverse knowledge backgrounds. For example, in high-tech entrepreneurial firms, the core team may be hesitant and reluctant to assemble potential partners with a knowledge background of business management because they are mostly engaged in technological solutions and development. It is perceived as unfair for them to share ownership and decision-making with potential partners who have not benefited the venture concept and operation. As such, our findings reveal that it is important for new ventures to foster a well-balanced team in terms of knowledge background, which will benefit creativity both individually and collectively. As a leading entrepreneur, it is advisable to implement a systematic assessment of team composition to identify complementary skills and capabilities. According to Der Foo et al. (2005), the recommendation of team composition has already been followed by venture capitalists who make funding decisions on the basis of the interdisciplinary characteristics of the entrepreneurial teams. In the same vein, it is also well-recommended for entrepreneurial incubators to underscore the importance of integrating heterogeneous partners into venture teams when providing support for potential leading entrepreneurs (Henneke and Lüthje, 2007).

Second, it is advisable for entrepreneurial educators, entrepreneurial incubators, funding institutions, and venture consultants to boost the possibility of heterogeneous team-building by recruiting potential partners from diverse knowledge backgrounds. Thus, for venture teams based on existing relationships, cultivating relationships with potential venture partners across knowledge disciplines seems reasonable prior to an actual new business start. Universities are believed to play a dominant role in facilitating cross-disciplinary team building. In this vein, university-based entrepreneurship education programs could not only facilitate the development of the entrepreneurial intention of students (Hou et al., 2019) but also provide a promising avenue to aid in cross disciplinary and work-experience contacts among students. Such entrepreneurship education programs could enhance the possibility for social interaction and integration among individuals from a variety of knowledge backgrounds and thus benefit the potential development of venture teams. In short, our findings indicate that it is necessary for entrepreneurship–fostering organizations to aid potential venture founders in assembling heterogeneous teams to attenuate and close gaps in their competency maps.

Third, the postfounding stage of established entrepreneurial teams is another story. In this case, in the short-term run, there are few chances to change the level of heterogeneity of venture team composition by recruiting additional partners with complementary knowledge backgrounds or by forced turnover of partners with redundant knowledge bases. In this case, given that the knowledge-based diversity of entrepreneurial teams may not be translated into TC automatically, it is recommended that team leaders should keep an eye on both team heterogeneity in terms of knowledge and the level of KS among team members to minimize the loss of creativity.

As indicated by our findings, the quality of KS mediates the relationship between ETKD and TC. This implies that established venture teams are necessary to be concerned with the level of KS, which may compensate for their lack of specific and complementary knowledge. Specifically, practices designed for team members to enhance idea sharing and divergent thinking are recommended (e.g., the devil's advocate approach; Park et al., 2018), which could facilitate the fostering of a participatory climate, thus benefiting TC potential.

Moreover, considering that our findings further reveal the sequential intermediating mechanism that underlies the link between team diversity and TC, venture team leaders should pay attention to individual-level members' cognitive processes (e.g., KS) and social contagion processes (e.g., individual creativity to TC). This indicates that ETKD could effectively foster team-level creativity on the condition that team members have higher-level signs of KS and individual creativity.

## Limitations and Future Directions

Although this study offers some interesting findings and implications, some limitations still need to be considered.

First, the relatively larger entrepreneurial team samples covering various industries in China should be taken into account. The confidence in the preliminary results could be enhanced by replicating this research with more samples of entrepreneurial teams. Despite this limitation, the current study is still considered valuable pilot research in that it provides a better understanding of how the heterogeneous composition of new entrepreneurial teams affects creativity both individually and collectively, which thus far has received limited attention.

Second, this study did not investigate some social group factors as intervening variables (e.g., the level of task conflict or the communication intensity of team members). For TMTs in mature firms, studies involving social group factors have generated promising results (Henneke and Lüthje, 2007). Due to the assumption that some social group factors (e.g., lack of communication among team members, severe affective conflicts) are verified to be less relevant for new venture teams, these social group variables were not included in this study. Nevertheless, it seems interesting for future research to further investigate the extent to which social group variables play a minor part in the relationship between ETKD and TC.

Third, drawing from Amabile's (1983) componential theory of creativity, factors that could facilitate individual learning lay the foundation for individual creativity. Extending this perspective, individual learning might be expected to relate to both skill acquisition and intrinsic motivation. And thus, it might motivate individuals to seek out opportunities for improving their creativity (Hirst et al., 2009). Additionally, building on theories of person-situation interactions (Tett and Burnett, 2003; Chen and Kanfer, 2006), contextual influence set the stage for generating creative outcomes. In this regard, team learning behavior, that is, collective problem solving and reflection (Edmondson, 1999) could be considered as one kind of contextual factors. Thus, future research is called on to further explore the diversity–creativity link by introducing learning-related factors (e.g., individual learning and team or organizational learning) to the theoretical model, and thus contribute to the creativity literature and broader organizational behavior field.

Finally, the dynamic effects of venture team composition were not considered in this study. Specifically, such effects posit that as a response to creative problems in generating and commercializing products or services, the composition of a given entrepreneurial team changes over time and may enhance the creativity and innovation of the team. With the growth of entrepreneurial teams, the running focus on the development of products or services might switch to operating procedures. In this sense, the composition of founding teams, initially dominated by homogenous members with backgrounds in science and technology, should correspondingly shift to heterogeneous members with backgrounds including management, finance, marketing, and legal aspects of ventures (Berry, 1996; Henneke and Lüthje, 2007). As such, future longitudinal studies are needed and are thus called on to further explore the dynamics of venture team composition in terms of knowledge diversity, which might shed light on the performance and competitive edge of entrepreneurial teams in the long run.

## Conclusion

This study aims to provide a deep understanding of the relationship between ETKD and TC, which aids in opening the “black box” of the direct effect of heterogeneous organizational demography on organizational outcomes in the context of entrepreneurial teams (Lawrence, 1997; Henneke and Lüthje, 2007). Furthermore, our findings provide evidence for the perspective that the relationship between entrepreneurial team characteristics and TC is mediated by intervening processes. Indeed, the diversity of entrepreneurial teams in terms of knowledge is an antecedent to both KS and TMC, which are conducive to the creativity of new ventures. In other words, KS and TMC fully mediate the link between ETKD and TC.

## Data Availability Statement

The raw data supporting the conclusions of this article will be made available by the authors, without undue reservation.

## Author Contributions

FH take charge of the research design, methodology, literature review, analysis and interpretation of data, as well as drafting and revising the manuscript. YS conceived the literature review, research design, and practice implication. MQ and LW contributed to the literature review, research design, and data collection. QW conceived the literature review and practice implication. All authors contributed to the article and approved the submitted version.

## Conflict of Interest

The authors declare that the research was conducted in the absence of any commercial or financial relationships that could be construed as a potential conflict of interest.

## Publisher's Note

All claims expressed in this article are solely those of the authors and do not necessarily represent those of their affiliated organizations, or those of the publisher, the editors and the reviewers. Any product that may be evaluated in this article, or claim that may be made by its manufacturer, is not guaranteed or endorsed by the publisher.

## Appendix

### The Questionnaire of Key Measurements

**Table:**

**Variables**		**Items**	**Measurement sources**
Entrepreneurial team knowledge diversity	ETKD1	Your entrepreneurial team members vary widely in their professional fields.	Campion et al., 1993; Jehn et al., 1999
	ETKD2	Your entrepreneurial team members have a variety of different educational backgrounds and experiences.	
	ETKD3	Your entrepreneurial team members have skills and abilities that complement each other.	
Knowledge sharing	KS1	I will always provide my manuals, methodologies, and models for members of my team.	Bock et al., 2005
	KS2	I will share my work reports and official documents with members of my team more frequently in the future.	
	KS3	I will try to share my expertise from my education or training with other team members in a more effective way.	
	KS4	I intend to share my experience or know-how from work with other team members more frequently in the future.	
	KS5	I will always provide my know-where or know-whom at the request of other team members.	
Team member creativity	TMC1	This team member seeks new ideas and ways to solve problems.	Tierney and Farmer, 2011
	TMC2	This team member identifies opportunities for new ways of dealing with work.	
	TMC3	This team member generates novel, but operable work-related ideas.	
	TMC4	This team member demonstrates originality in his/her work.	
Team creativity	TC1	Your team often comes up with new and practical ideas to improve performance.	George and Zhou, 2001
	TC2	Your team suggests new ways to achieve goals or objectives.	
	TC3	Your team develops adequate plans and schedules for the implementation of new ideas.	
	TC4	Your team comes up with creative solutions to problems.	
	TC5	Your team often has a fresh approach to problems.	
	TC6	Your team suggests new ways of performing work tasks.	
